# Supplementary material for: An Update on the Management of Acute High-Risk Pulmonary Embolism
Source: J Clin Med. 2022 Aug 17;11(16):4807. doi: 10.3390/jcm11164807 (PMC9409943; doi:10.3390/jcm11164807)
Supplement: Supplementary file 1 [file jcm-11-04807-s001.zip › jcm-1812631-supplementary.pdf]

Supplemental material File S1. Detailed search terms.

An update on the management of acute high-risk pulmonary embolism

eAppendix

Pubmed database history and Search Details

Search	Query	Results
#1	Search: **((pulmonary embolism[Title]) AND (high-risk[Title] OR high risk[Title] OR massive[Title])) AND (("2000/01/01"[Date - Publication] : "2022/05/31"[Date - Publication]))** Sort by: **Most Recent**	1,025

Ovid search history

	Searches	Results
1	pulmonary embolism.m_titl.	7300
2	limit 1 to yr="2000 -Current"	6217
3	(high-risk or high risk or massive).m_titl.	32526
4	limit 3 to yr="2000 -Current"	28308
5	2 and 4	508

Cochrane data search histoy

(high risk OR high-risk OR massive) "pulmonary embolism"

Cochrane Library publication date from Jan 2000 to May 2022

in Cochrane Reviews (Word variations have been searched). 373 results
